# *De Novo* Design of Protein-Binding Peptides by Quantum Computing

**DOI:** 10.1021/acs.jctc.5c00768

**Published:** 2025-09-29

**Authors:** Lars Meuser, Alexandros Patsilinakos, Pietro Faccioli

**Affiliations:** † Dipartimento di Fisica, 695711 Università di Milano-Bicocca, Piazza della Scienza 3, 20126 Milano, Italy; ‡ Sibylla Biotech S.p.A., Via Lillo del Duca 10, 20091 Bresso, Italy; § 9305 INFN Sezione di Milano-Bicocca, Piazza della Scienza 3, 20126 Milano, Italy

## Abstract

Physics-based approaches to *de novo* drug design involve the simultaneous exploration of vast chemical and conformational spaces. The rapid development of quantum computing technologies offers a promising perspective to efficiently tackle this challenge. In this work, we focus on peptide design and present a multiscale framework that combines classical and quantum computing to optimize amino acid sequences and predict binding poses at atomic resolution. We illustrate our scheme by designing binders for several protein targets, and we contrast the performance of the D-Wave quantum annealer with that of an industry-grade classical optimizer. To assess our results, we compare the designed sequences and the corresponding poses with those available in a data set of experimentally characterized peptide binders.

## Graphical abstract

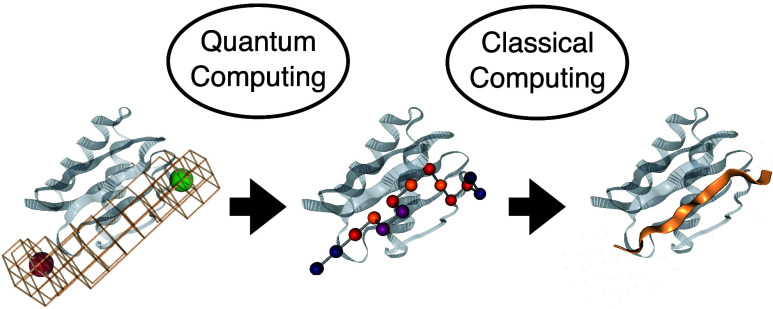

## Introduction

Contemporary drug discovery research increasingly resorts to *in silico* approaches to drastically reduce the time and cost of developing drug candidates. For example, a customary approach in structure-based drug discovery consists of identifying hit candidates by performing virtual screening campaigns over libraries containing as many as 10^10^ compounds. The selected molecules must then be optimized to improve affinity, solubility, and deliverability and to reduce toxicity. This procedure is very time-consuming and expensive and involves several iterations of computer simulations and experiments.

An alternative and potentially more efficient approach is one in which hit candidates are designed *de novo*, taking into consideration the specific chemical environment provided by the binding pocket (for a recent review, see, e.g., ref 1). Algorithms for this purpose may assemble preselected molecular fragments^2,3^ or update existing molecular structures with random mutations and crossovers.^4,5^ In recent years, several successful deep learning (DL) methods have been developed using a wide range of different neural network architectures (see, e.g., refs 6–20 and references therein). At the same time, generative DL schemes still struggle to produce molecules with high affinity^21^ and synthesizability.^22^ Importantly, most DL schemes require target-specific data and are inherently biased toward generating molecules within the local chemical space of that data. Exploration is further limited by the fact that learned scoring functions often lose accuracy when evaluating poses that differ from those seen during training. While active learning schemes may help to tame this problem,^6–8^ they come at a much higher computational cost.

In contrast to DL-based schemes, “bottom-up” approaches based on modeling the statistical physics of the protein–ligand complex do not rely on training data sets. However, they require to explicitly account for the intra- and intermolecular interactions. Furthermore, finding optimal ligands poses a formidable optimization problem, as it involves simultaneously exploring chemical and conformational space.

The rapid development of quantum hardware raises the question of whether quantum computing can be harnessed to solve the optimization problem involved in *de novo* drug design. In this context, a particularly attractive feature of quantum computers is that, in principle, they enable the exploration of an exponentially large, combinatorially complex search space by exploiting quantum tunneling and superposition. At the same time, several key tasks, including the mathematical representation of three-dimensional macromolecular structures and the implementation of classical force fields, are carried out more efficiently on classical computers.

In this work, we develop a physics-based scheme to tackle the *de novo* drug design problem, integrating classical and quantum computing to exploit their respective advantages. As a first step in this direction, we focus on peptide-based drug design, optimizing both the sequence and binding pose to maximize the binding affinity. Peptide binders are an emerging class of drugs with applications in diverse therapeutic areas such as metabolic disorders and cancer.^23–25^ Compared to small-molecule drugs, they are less toxic and more readily synthesizable but have limited membrane permeability and are metabolized faster. From a computational perspective, peptides have a simpler topology and a smaller number of building blocks compared to small molecules. Furthermore, empirical estimates of the affinity between the different amino acid types^26^ can be used to estimate the interaction energy at a coarse-grained level of resolution.^27^ This enables us to employ a multiscale approach, where the simultaneous exploration of chemical and conformational space is carried out at the coarse-grained level, reducing the number of binary variables required and allowing us to solve the optimization problem on the D-Wave quantum annealer. The corresponding binding pose is then determined with a classical computer at full atomic resolution.

To validate our approach, we develop a statistical analysis to compare the predicted sequences and binding poses with those available in experimental data sets. We also compare the results from D-Wave’s hybrid classical-quantum solver with those obtained by an industry-grade classical solver.

## Results

### Statistical Physics Formulation of the Ligand Design Problem

From a statistical physics perspective, the general problem of identifying optimal ligands for a given fixed target protein *P* can be formulated as

1 $$\max_{\Gamma ,\Sigma }\frac{\exp\left(-\frac{U\left(\Gamma ,\Sigma ;P\right)}{k_{B}T}\right)}{\sum_{P^{\prime }}\sum_{\Gamma^{\prime }}\exp\left(-\frac{U\left(\Gamma^{\prime },\Sigma ;P^{\prime }\right)}{k_{B}T}\right)}$$

In this expression, *U*(Γ, Σ; *P*) denotes the interaction free energy of the system consisting of a ligand of chemical composition Σ in a configurational state Γ and a protein *P* in its native conformation. We stress that this free energy should also include the implicit contribution of the internal degrees of freedom. For example, for solvation effects in implicit solvent models or for the entropy associated with the side-chain configurations, in coarse-grained models, only the backbone arrangement is specified.

We also note that eq 1 does not account for induced-fit interactions and, in general, for any effect associated with the conformational entropy of the target. In the section “Improvements required for accurate peptide design”, we discuss a possible strategy to overcome this limitation.

The summation over all possible protein targets in the denominator ensures that the designed ligands selectively maximize the affinity for the given target. We can equivalently reformulate the optimization problem eq 1 as

2 $$\min_{\Gamma ,\Sigma }\left(U\left(\Gamma ,\Sigma ;P\right)-G\left(\Sigma \right)\right)$$

where

3 $$G\left(\Sigma \right)\equiv -k_{B}T\ln\sum_{P}\sum_{\Gamma }\exp\left(-\frac{U\left(\Gamma ,\Sigma ;P\right)}{k_{B}T}\right)$$

is interpreted as the free energy associated with a given chemical structure Σ. Unfortunately, computing *G*(Σ) is a formidable task because it involves accounting for all possible protein targets and, for each of them, summing the Boltzmann factors of all ligand configurational states. To reduce computational costs, we approximate its cumulant expansion truncated to the lowest order and obtain

4 $$\min_{\Gamma ,\Sigma }\left(U\left(\Gamma ,\Sigma ;P\right)-\langle U\left(\Sigma \right)\rangle_{0}\right)$$

where $\langle U\left(\Sigma \right)\rangle_{0}\equiv \frac{1}{N_{S}}\sum_{P}\sum_{\Gamma }U\left(\Sigma ,\Gamma ;P\right)$ and $N_{S}\equiv \sum_{P}\sum_{\Gamma }1$ . According to condition eq 4, the optimal ligand is the one that minimizes the binding energy with the given target *P*, relative to its average interaction with proteins.

### Designing a Simplified Coarse-Grained Model

Let us now restrict our focus to the case in which the ligand is a peptide. In the following, we develop a coarse-grained mathematical representation of the peptide’s primary sequence Σ, the chain’s conformational state Γ, and the interaction free energy *U*(Γ, Σ; *P*) that can be encoded on a collection of interacting two-level quantum systems (qubits).

We represent amino acids with single beads and group them into *D* with different chemical families. Furthermore, we discretize the peptide’s conformational space by introducing a square lattice that fills the pocket *P* of the target protein (see the leftmost panel in Figure 1). The lattice spacing is set to match the length of the peptide bond (0.38 nm) so that each configurational state of the peptide in the pocket can be identified with a self-avoiding path on the lattice. In contrast, the protein’s three-dimensional structure is represented using an off-lattice continuous model.

**Figure 1:**
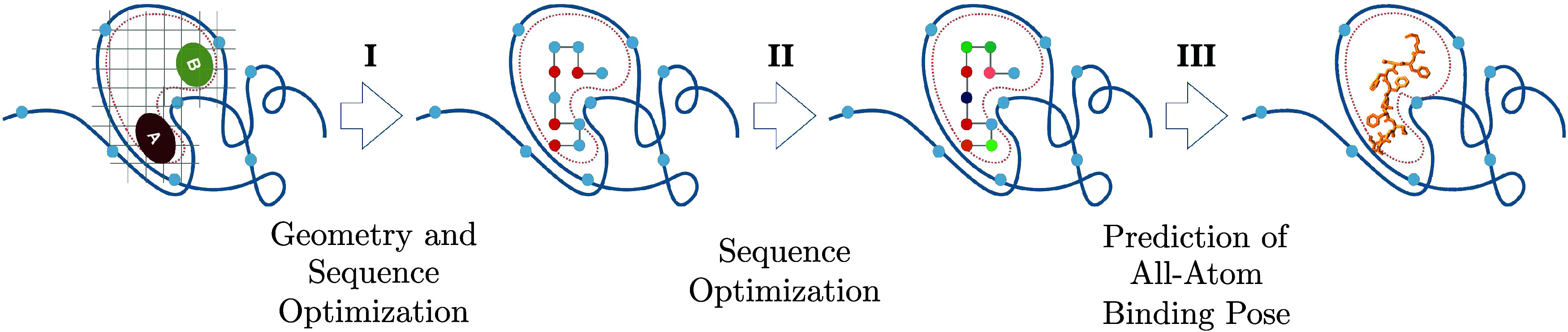
Schematic depiction of the workflow. (I) Coarse-grained peptide connecting regions A and B is generated via (quantum) minimization of the problem Hamiltonian (eq 12). (II) Configuration is frozen, and the sequence is optimized in higher resolution by a second (quantum) minimization step. III: Classical molecular docking simulation predicts the all-atom off-lattice representation of the previously generated sequence.

To derive an expression for the interaction energy *U*(Σ, Γ; *P*), we resort to the Miyazawa–Jernigan knowledge-based potential, first introduced in.^26^ Specifically, we follow the formulation proposed by Kim and Hummer,^27^ which includes Lennard–Jones (LJ) pairwise interactions between different amino acids in the protein and in the peptide. To define such an interaction, we first introduce two 20 × 20 matrices of parameters, with entries indexed by *i*, *j* ∈ {1, ..., 20}

1. An energy matrix ε̂ with entries
5 $$\varepsilon_{\mathrm{ij}}=\lambda \left(e_{\mathrm{ij}}-e_{0}\right)$$
  where λ = 0.159 provides an overall scale, *e*_*ij*_ is the entry of the original Miyazawa–Jernigan matrix reported in ref 26, and *e*_0_ = −2.27 *k*_B_ *T* is an overall energy offset.
2. An interaction range matrix with entries
6 $$\sigma_{\mathrm{ij}}=\frac{\sigma_{i}+\sigma_{j}}{2}$$
  where σ_*i*_ denotes the van der Waals (vdW) diameter of the amino acid of type *i* (the numerical values are reported in ref 27).

The LJ interaction between an amino acid of type *i* and one of type *j* at a relative distance *r* is given by

7 $$u_{\mathrm{ij}}\left(r\right)=\{\begin{array}{ll} 4\left|\varepsilon_{\mathrm{ij}}\right|\left[{\left(\frac{\sigma_{\mathrm{ij}}}{r}\right)}^{12}-{\left(\frac{\sigma_{\mathrm{ij}}}{r}\right)}^{6}\right] & \varepsilon_{\mathrm{ij}}<0\\ 4\varepsilon_{\mathrm{ij}}\left[{\left(\frac{\sigma_{\mathrm{ij}}}{r}\right)}^{12}-{\left(\frac{\sigma_{\mathrm{ij}}}{r}\right)}^{6}\right]+2\varepsilon_{\mathrm{ij}} & \epsilon_{\mathrm{ij}}>0,r<r_{\mathrm{ij}}^{0}\\ -4\varepsilon_{\mathrm{ij}}\left[{\left(\frac{\sigma_{\mathrm{ij}}}{r}\right)}^{12}-{\left(\frac{\sigma_{\mathrm{ij}}}{r}\right)}^{6}\right] & \epsilon_{\mathrm{ij}}>0,r\geq r_{\mathrm{ij}}^{0} \end{array}$$

where *r*_*ij*_^0^ ≡ 2^1/6^σ_*ij*_. We set a cutoff for all LJ interactions at 8.5 Å, a choice in line with the values adopted in binary contact maps based on *C*_α_ distances.

The model defined so far distinguishes among the 20 different types of naturally occurring amino acids. However, the chemical alphabet of amino acids is known to be redundant^28^ as multiple residues share similar physicochemical properties, such as, e.g., electric charge, polarity, and hydrophobicity. This redundancy can be exploited to develop an even coarser-grained approach in which amino acids are grouped into *D* < 20 families. Theoretical studies have suggested that optimal grouping should include 5–10 families^28,29^ to preserve most of the structural information.

Deriving this new representation amounts to mapping the 20 × 20 energy matrices *ê* and σ̂ onto *D* × *D* effective matrices *ê*′ and σ̂′. The assignment of the 20 amino acids to the *D* clusters can be done by finding the mapping *a*(*i*) ∈ {1, ..., *D*} that minimizes the loss function

8 $$L=\sum_{i,j=1}^{20}{\left(e_{\mathrm{ij}}-e_{a\left(i\right)a\left(j\right)}^{\prime }\right)}^{2}$$

The entries of the corresponding effective (clustered) interaction matrix are defined by a mean over the elements of the clusters, i.e.,

9 $$e_{\mathrm{ij}}^{\prime }=\frac{1}{N}\sum_{k,l=1}^{20}e_{\mathrm{kl}}\delta_{a\left(k\right),i}\delta_{a\left(l\right),j}$$

with N normalizing over the number of elements included in the two sets. Similarly, the effective interaction range matrix is obtained by averaging over the elements of a cluster, i.e.,

10 $$\sigma_{I}^{\prime }=\frac{1}{N}\sum_{k=1}^{20}\delta_{a\left(k\right),I}\sigma_{k}$$

We checked that this clustering procedure generates groups with similar physicochemical properties. For example, by choosing *D* = 2, we obtain a bipartition of the amino acids that reflects the hydrophobicity of the residues.

To solve the design problem in eq 4, we need to evaluate the average interaction of the peptide Σ with proteins, ⟨*U*(Σ)⟩_0_. To estimate this term using a manageable amount of qubit resources, we introduce a mean-field approximation

11 $$\langle U\left(\Sigma \right)\rangle_{0}\approx N_{c}\sum_{n=1}^{l_{\Sigma }}\sum_{j=1}^{20}f_{j}\epsilon_{i\left(n\right)j}$$

In this equation, *l*_Σ_ is the peptide length and *i*(*n*) is the type of the amino acid at position *n* along the chain. *f*_*j*_ is the relative frequency of the amino acid type *j* on the surface of a typical protein, obtained from ref 30, and $N_{c}$ is the average number of contacts that a peptide residue forms with the amino acids on a typical protein surface (see also the Supporting Information (SI) for details). We note that in eq 11 the average interaction that the amino acid sequence Σ forms with typical protein surfaces is estimated by the interaction it would form on a fictitious average protein surface.

### Quantum Encoding of the Design Optimization Problem

To be able to use the D-Wave quantum annealer to solve the peptide binder design problem, we encode condition eq 4 as a Quadratic Unconstrained Binary Optimization (QUBO) problem. This requires mapping favorable peptide sequences and binding poses onto the low-energy states of a suitably defined quantum Hamiltonian, *Ĥ*. To this end, it is convenient to first establish a QUBO encoding based on classical binary variables (bits) and then to promote the formulation to the quantum level, replacing them with qubits.

We introduce a collection of binary variables *q*_*i*_^(*k*)^ ∈ {0,1} at each grid point *i*, which are set to 1 if the site *i* is occupied by a residue of type *k* ∈ {1,..., *D*}. Additional binary variables *q*_*ij*_ denote the formation of a chemical bond between the residues at the neighboring grid points *i* and *j*. Lastly, we resort to a set of ancillary variables, *q*_*ij*_^(*k*)^, that are required to ensure that the Hamiltonian is, at most, quadratic in the binary variables.

The overall structure of our classical QUBO Hamiltonian consists of several terms

12 $$H=H_{\mathrm{int}}+H_{\mathrm{ext}}+H_{\mathrm{anc}}+H_{\mathrm{occ}}+H_{\mathrm{path}}+H_{\mathrm{chain}}$$

The first two terms, *H*_int_ and *H*_ext_, represent the interactions of the peptide with itself and with the residues in the pocket, respectively. In particular, the latter is given by

13 $$H_{\mathrm{ext}}=\sum_{i}^{\prime }\sum_{k=1}^{D}\left(E_{i}^{\left(k\right)}-E_{0}^{\left(k\right)}\right)q_{i}^{\left(k\right)}$$

where ∑_*i*_^′^ indicates the sum over grid points. *E*_*i*_^(*k*)^ is the (precomputed) energy an isolated amino acid of type *k* would experience at the lattice site *i* due to the interaction with the target protein’s amino acids in the pocket. *E*_0_^(*k*)^ accounts for condition eq 4, corresponding to the average interaction this isolated amino acid forms with a generic protein surface, and it is evaluated according to eq 11. *H*_int_ is the Hamiltonian accounting for nonbonded intrachain interactions within the peptide and reads

14 $$H_{\mathrm{int}}=\sum_{i.j}^{\prime }\sum_{k,l=1}^{D}u_{\mathrm{kl}}\left(r_{\mathrm{ij}}\right)\left(q_{i}^{\left(k\right)}-q_{\mathrm{ij}}^{\left(k\right)}\right)q_{j}^{\left(l\right)}$$

where *r*_*ij*_ denotes the Euclidean distance between lattice sites *i* and *j*. The ancillary variables *q*_*ij*_^(*k*)^ are defined on neighboring sites *i* and *j* only. For neighboring sites, they are set to 1 when the residue of type *k* at the site *i* is involved in a chemical bond with a residue of any type at the neighboring site *j*, i.e., if *q*_*ij*_ = *q*_*i*_^(*k*)^ = 1. This consistency condition is enforced by the Hamiltonian

15 $$H_{\mathrm{anc}}=A\sum_{<i,j>}^{\prime }\sum_{k=1}^{D}\left({3q}_{\mathrm{ij}}^{\left(k\right)}+q_{j}^{\left(k\right)}q_{\mathrm{ij}}-{2q}_{j}^{\left(k\right)}q_{\mathrm{ij}}^{\left(k\right)}-2q_{\mathrm{ij}}q_{\mathrm{ij}}^{\left(k\right)}\right)$$

where ∑_⟨*i*, *j*⟩_^′^ represents the sum over neighboring lattice sites and *A* is a positive constant that sets the overall energy penalty for violating the constraint. Note that with this definition, the factor (*q*_*i*_^(*k*)^ – *q*_*ij*_^(*k*)^) excludes the interactions between covalently bonded amino acids.

The term *H*_occ_ in eq 12 ensures that each grid point is occupied by at most one amino acid and reads

16 $$H_{\mathrm{occ}}=A\sum_{i}^{\prime }\sum_{k\neq l}^{D}q_{i}^{\left(k\right)}q_{i}^{\left(1\right)}$$

*H*_path_ is defined to enforce the peptide’s linear topology, i.e., to ensure that the internal residues along the chain are bonded to exactly two neighbors, while those at the terminals form a single bond

17 $$H_{\mathrm{path}}=A\left(h_{t}+h_{s}+h_{r}\right)$$

18 $$h_{s}={\left(1-\sum_{k=1}^{D}q_{s}^{\left(k\right)}\right)}^{2}+{\left(\sum_{k=1}^{D}q_{s}^{\left(k\right)}-\sum_{j\in <s,j>}^{\prime }q_{\mathrm{sj}}\right)}^{2}$$

19 $$h_{t}={\left(1-\sum_{k=1}^{D}q_{t}^{\left(k\right)}\right)}^{2}+{\left(\sum_{k=1}^{D}q_{t}^{\left(k\right)}-\sum_{j\in <t,j>}^{\prime }q_{\mathrm{tj}}\right)}^{2}$$

20 $$h_{r}={\sum_{j\neq s,t}^{\prime }\left(2\sum_{k=1}^{D}q_{r}^{\left(k\right)}-\sum_{j\in <r,j>}^{\prime }q_{r,j}\right)}^{2}$$

The terms (1 – ∑_*k* = 1_^*D*^ *q*_*s*(*t*)_^(*k*)^)^2^ in eqs 18 and 19 specify the location of the chain end points, while (2∑_*k* = 1_^*D*^ *q*_*r*_^(*k*)^ – ∑_*j*∈⟨*r*,*j*⟩_^′^ *q*_*rj*_)^2^ takes care of the chain’s continuity requirement. We note that, in the limiting case of just one chemical species, *H*_path_ coincides with the QUBO Hamiltonian introduced in ref 31 to identify paths connecting two given nodes in a discrete network.

For the Hamiltonians eqs 16–18 to encode hard constraints, the energy scale A must be large compared with all soft interactions. Under this condition, low-energy configurations of Hamiltonian eq 18 are those representing a linear chain that connects lattice sites *s* and *t*.

In addition to the chain, some of these states may also include topologically disconnected circularized peptides, which may be removed in postprocessing. Alternatively, they can be suppressed by introducing additional penalty terms in *H*, specifically designed to penalize circular structures.^32,33^ Finally, spurious polymer rings are also suppressed when the total number of bonds (chain length *L*_0_) is comparable to the shortest distance between the lattice sites *s* and *t. L*_0_ can be fixed by introducing an additional constraint

21 $$h_{\mathrm{chain}}=\omega {\left(L_{0}-\sum_{<i,j>}^{\prime }q_{\mathrm{ij}}\right)}^{2}$$

The factor *w* can be tuned according to the desired tolerance on the length of the generated chains. To ensure a given fixed length, *w* needs to be set at a value comparable to *A*. In contrast, the choice $w∼\frac{A}{L_{0}^{2}p^{2}}$ allows for generating chains with slightly different lengths, with relative fluctuations of the order of *p* percent, i.e., *L* ∈ [*L*_0_(1 – *p*), *L*_0_(1 + *p*)].

The advantage of the present formulation over the approach taken in ref 34 is that it does not require fine-tuning of the weights and allows control over the magnitude of the fluctuations in the length of the generated linear peptides. However, a disadvantage is the introduction of all-to-all connectivity between the bond variables, potentially making the optimization problem harder to tackle, with both classical and quantum computers.

In the Supporting information we show that, for a cubic lattice of dimensions *L*_*x*_, *L*_*y*_, *L*_*z*_, the total number of binary variables required is

22 $$N_{\mathrm{qubits}}∼L_{x}L_{y}L_{z}\left(4D+3\right)$$

### Peptide Design Algorithm

Our peptide design algorithm operates according to the following multistep procedure, which is schematically illustrated in Figure 1

1. Using the binary encoding described in “Quantum Encoding of the Design Optimization Problem” and the coarse-grained model defined in “Designing a Simplified Coarse-Grained Model”, we perform a simultaneous optimization of both the chain’s primary sequence and its binding pose. To meet the limitations on the maximum number of qubits available on the existing quantum computing hardware, in this phase, we resort to a clustering algorithm to restrict the chemical alphabet to *D* families, with 5 ≲ *D* ≲ 10.
2. The location of the amino acids on the lattice obtained after the minimization in the previous step is held fixed, enabling more qubit resources to become available for a second, more refined optimization of the primary sequence that includes the full range of 20 amino acids. Fixing the conformation allows us to drop the ancillary qubit in *H*_int_, as well as the terms *H*_anc_, *H*_path_, and *H*_chain_.
3. The selected chain sequence Σ is then passed to a state-of-the-art docking software, which returns the off-lattice, atomistically detailed binding pose. In this work, we resorted to Autodock CrankPep (ADCP),^35^ a specialized version of the Autodock software package designed for peptide docking.

In Figure 2, we summarize the computational workflow of our design algorithm.

**Figure 2:**
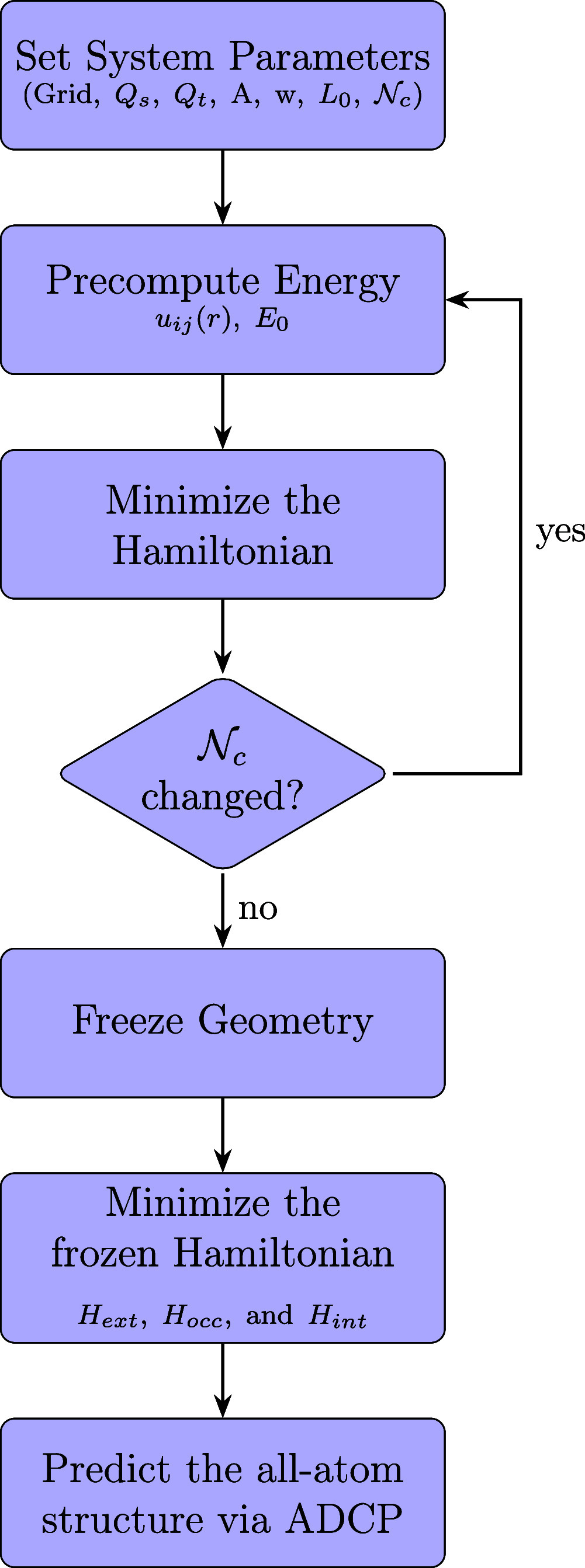
Flowchart representing the computational workflow of our hybrid quantum-classical algorithm. $N_{c}$ is defined once for a given force field and may be slightly adjusted during simulations.

### Application to Protein–Peptide Complexes

In the following, we report several applications of our peptide design algorithm, and we provide a first assessment of its accuracy based on experimentally resolved protein–peptide complexes. Note that the minimization in this case was performed on a classical device. Nevertheless, all system sizes are compatible with the capabilities of the current quantum hardware. For the protein–peptide complex (3BRL), we performed a comparison between results obtained on an actual quantum device and those obtained classically; see “Classical vs quantum optimization” for details.

#### Illustrative Toy Model

As a first example, we focus on the optimization step of the chemical and conformational peptide’s structure using the simplest possible model, in which the chemical alphabet is reduced to just two types of amino acids. Even though the couplings of this model are obtained by clustering the full Miazawa–Jerningan matrix, they essentially provide a realization of the celebrated HP model.^36,37^ Note, however, that our amino acid clustering algorithm misclassifies tryptophan, tyrosine, and histidine, residues that exhibit both hydrophobic and polar character (e.g., tyrosine has a hydrophobic aromatic ring, while at the same time, it carries a polar hydroxyl group). The clustering scheme also misclassifies proline, which, despite being hydrophobic, is often solvent-exposed due to its role as a helix breaker. Our aim is to explore how varying the parameter *w* in eq 21 affects the design and to assess whether the resulting sequences are chemically plausible. In this simulation, we set the chain length parameter to *L*_0_ = 10 and the hard constraint parameter to *A* = 10.

Figure 3 shows the two lowest-energy designs obtained using Gurobi for four different values of the chain stiffness parameter *w*, from left to right: *w* = 10, *w* = 0.625, *w* = 0.28, and *w* = 0.1. The average number of contacts $N_{c}$ was 7.9, 8.0, 7.5, and 7.2, while the length of the peptides increased from 10 to 12, 14, and 16 residues. Note that the algorithm correctly places hydrophobic residues near the pocket’s core and hydrophilic residues in the outer, solvent-exposed regions. As expected, decreasing *w* allows the algorithm to lower the total energy by increasing the chain length, thereby slightly relaxing the *L* = 10 constraint (see rightmost structures). These values of *w* correspond to $w=\frac{A}{L_{0}^{2}p^{2}}$ with *p* = 0.1, 0.2, 0.6, and 1.0, which, in principle, should allow for chains of length 11, 12, 16, and 20, respectively, as discussed in “Quantum encoding of the design optimization problem”. The observed lengths are slightly shorter but nonetheless confirm that the Hamiltonian follows the expected trend.

**Figure 3:**
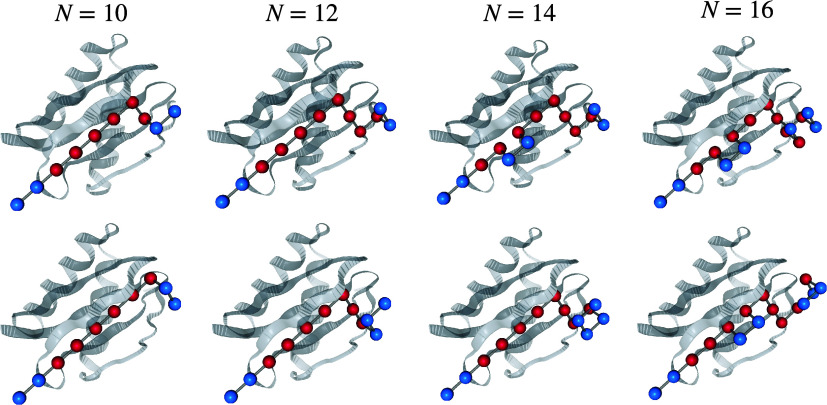
Generated peptides of varying length for *D* = 2 chemical species. The peptides were generated with *L*_0_ = 10 and varying chain stiffness *w*. Each column displays the two lowest-energy configurations for the corresponding peptide length. Lower values of *w* (i.e., reduced stiffness) result in longer, more flexible chains. Blue beads represent polar residues, while red beads represent hydrophobic amino acids. Note that the algorithm places hydrophobic residues closer to the pocket’s core, while polar residues are more exposed to the solvent.

Building on this successful toy model, we extend our approach to more realistic scenarios by expanding the amino acid alphabet and combining combinatorial optimization with docking to obtain fully resolved three-dimensional structures. Even though many experimentally resolved structures for protein–peptide complexes are available as PDB files, exploiting this information to assess the accuracy of our peptide design algorithm is not straightforward. Indeed, since the peptides’ chemical space grows exponentially with the number of residues, it is extremely unlikely for any design algorithm to yield the sequences found in any of the available protein–peptide PDB entries. Likewise, the chains in the experimentally resolved complexes are not necessarily those with the highest binding affinity. To overcome this problem and to meaningfully assess our algorithm, we devised two independent statistical analyses, which focus on the structure of the binding pose and on the peptides’ primary structure.

#### Structure-Based Validation

In principle, the quality of our algorithm may be assessed by comparing the binding free energies of the designed peptides to those of the peptide in the PDB structure, ensuring that they are much larger than those of randomly generated peptides. In practice, accurate free-energy calculations are computationally expensive and require precise knowledge of binding modes, which we do not have. Furthermore, experimental validation is beyond the scope of this initial model. For validation, we used ADCP, the same tool we utilized earlier in our pipeline for all-atom structure prediction. While ADCP provides estimates of absolute binding free energies, these can be affected by large systematic errors. Nevertheless, ADCP performs well in structural predictions for a ligand’s binding pose and in ranking alternative binding poses of the same ligand based on their estimated relative binding free energy.^38^ These two features can be leveraged to devise a precision–recall analysis that assesses the quality of the designed peptides by comparing structural predictions rather than absolute binding free energies. To this end, we assume that the key interactions made by the peptide found in the PDB structure (referred to as the peptide’s native contacts) are universal, i.e, common to all ligands that bind to the given pocket. In other words, good binders are assumed to form a large fraction of native contacts, *f*_nat_. ADCP generates a ranked list of predicted binding poses based on a proxy of the free energy.

To assess our algorithm, for each generated sequence, we used ADCP to produce a list of possible binding poses and checked if the poses with the largest values of *f*_nat_ were ranked high on this list. To quantify this test, we conducted a precision–recall analysis, marking poses with *f*_nat_ > 0.5 as positive, a criterion also used in the Critical Assessment of Predicted Interactions (CAPRI).^39^

We considered three different protein–peptide complexes (PDB codes: 3BRL, 4DS1, 3BFW) taken from the LeadsPep data set,^40^ with peptides containing 10 and 11 amino acids. First, we removed the peptides from the PDB files and set up a lattice in the corresponding protein pocket, placing it in regions within 7.6 Å of the C_α_ atoms in the experimental binding pose. We also excluded points that were within a distance of 1.5 Å of the receptor. Next, we chose the lattice sites *s* and *t*, assigning them to be the grid points closest to the end points of the experimentally bound peptide. A more general and computationally expensive approach would be to compare the results obtained while also varying the locations of the end point sites *s* and *t*, retaining the best-scoring choice.

Following the procedure outlined in Figure 1, we first performed the simultaneous optimization of the sequence and configuration space with *D* = 5. The parameter for the hard constraints was set to *A* = 20, and the parameter governing the chain flexibility was set to *w* = *A*. The chain length *L*_0_ was set to match the number of amino acids in the known peptides, i.e., 10 and 11. The average number of contacts, $N_{c}$ , was found to be 7.5, 6.5, and 7.9. After freezing the top-scoring geometry, we performed the sequence optimization with the full set of *D* = 20 natural amino acids. The best-scoring element was then passed to ADCP, generating 100 ranked poses of this sequence in an off-lattice all-atom representation.

In general, the quality of these predictions depends on three main factors: (i) the accuracy of our coarse-grained energy model, (ii) the efficiency of the quantum optimization algorithm in identifying high-affinity sequences for the given pocket, and (iii) the reliability of the docking software in predicting the correct off-lattice binding pose. To disentangle these factors, we used ADCP to perform two additional sets of calculations:

- Predicting binding poses for 30 randomly generated sequences.
- Redocking the peptide present in the experimentally resolved protein–peptide complex.

The results of our precision–recall analysis for all three protein–peptide complexes are reported in Figure 4. As expected, the redocking of the original peptides yields a high area under the precision recall curve (AUPRC) score (orange vertical line) in all three cases. Conversely, the results based on docking randomly generated peptides (green histograms) show a broad distribution, with an average AUPRC score close to 0.4. The AUPRC score of the peptides designed with our algorithm (blue vertical lines in Figure 4) is significantly closer to the experimental pose than the average AUPRC score of the randomly generated peptides. In particular, for proteins 3BRL and 4DS1, the design algorithm yields remarkably good results, close to the ideal limit of our algorithm, set by the redocking curve. However, we note that while the average random peptide performs significantly worse, some random peptides perform comparably well.

**Figure 4:**
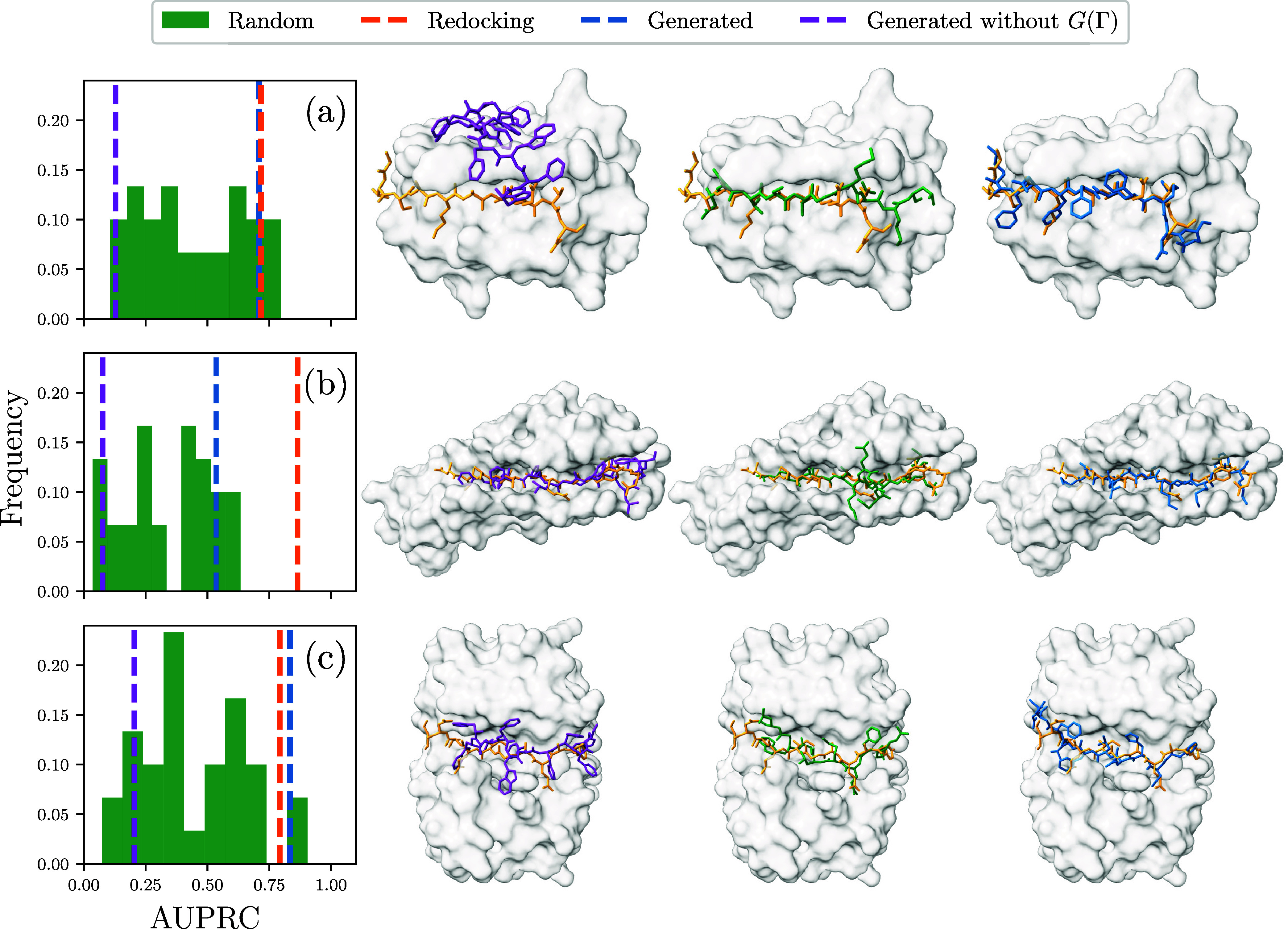
Left: The area under the precision–recall curve for docking simulations of the PDB entries (a) 3BRL, (b) 3BFW, and (c) 4DS1. For each simulation, the scores of 30 random peptides (green) were compared to those of the previously removed peptide (orange), a generated peptide optimized without accounting for the sequence free energy (purple), and a generated peptide optimized accounting for it (blue). A higher score indicates better binding to the pocket in the framework of the docking validation (see the main text and the Supporting information). Right: The top-ranked structures of the simulations yielded the results on the left. For the random ones, the structure closest to the mean fraction of native contacts was used.

We recall that our algorithm accounts for the peptide’s average interactions using a mean-field approximation (eq 11), enforcing the selectivity of the designed peptide to the given target. To investigate how our results are affected by this condition, we performed additional peptide design runs in which we neglected this factor, i.e., leaving out *E*_0_^(*k*)^ in eq 13. We found significantly worse results, as shown by the purple vertical lines. The fact that the resulting sequences perform even worse than the randomly generated ones suggests that neglecting ⟨*U*(Σ)⟩_0_ introduces a systematic error. This effect is explained by the coarse-grained interaction energy being the most attractive between hydrophobic residue pairs. Hence, optimizing the sequence to minimize only *U*(Γ, Σ; *P*) yields hydrophobic sequences not specifically designed to match the chemical environment provided by the pocket.

#### Sequence-Based Validation

For a sequence-based validation of our design algorithm, we resorted to a data set containing 111 peptides that bind to a specific pocket of the LC8 protein, a molecular hub protein, which takes part in cell homeostasis. As discussed in detail in ref 41,42 , the peptides in the database interact with the LC8 pocket via an 8-amino acid recognition motif. We used our algorithm to design 50 different eight-residue peptides predicted to bind to the same pocket. 3BRL is a protein–peptide complex consisting of a peptide that binds to LC8. Therefore, aside from setting *L*_0_ = 8 and choosing the end points according to the binding motif, the parameters for the simulation were kept identical. The goal of our approach was to assess the quality of our approach by comparing the primary sequences of the designed peptides with those in the experimental database.

As already mentioned, the chemical space of the designed peptide chains is huge, so it is unlikely that any design algorithm will generate sequences present in the data set. In “Designing a simplified coarse-grained model”, we exploited the redundancy of the amino acid alphabet to develop a coarse-grained model in which the 20 amino acid types were grouped into 5 ≲ *D* ≲ 10 families. The same procedure enabled us to compare the designed and experimentally available sequences: We aim to identify correlations among the amino acid families found at different positions. In particular, the analysis reported in Figure 5 was performed by grouping the amino acids into 5 families. Each of the eight histograms corresponds to one position in the binding motif, while the bars represent the relative frequency of the members of each of the 5 families: the blue (orange) bars refer to the relative population in the designed peptides (experimental data set). In comparing these two distributions, one should keep in mind that the experimental data set may not provide an unbiased sample and might not represent the most optimal molecules for binding to the protein pocket. In spite of these limitations, the comparison between the distributions can still provide at least a qualitative assessment. Overall, we find a good correlation between the histograms corresponding to the different data sets. For example, at 6 out of the 8 positions (namely 1, 2, 4, 6, 7, and 8), the most frequently occurring amino acid family in the experimental data set is among the two most frequently occurring families in the designed data set. Positions 2, 6, and 8 show particular correlations between the data sets, suggesting that our design code can recognize which amino acid type is required at this position to promote binding.

**Figure 5:**
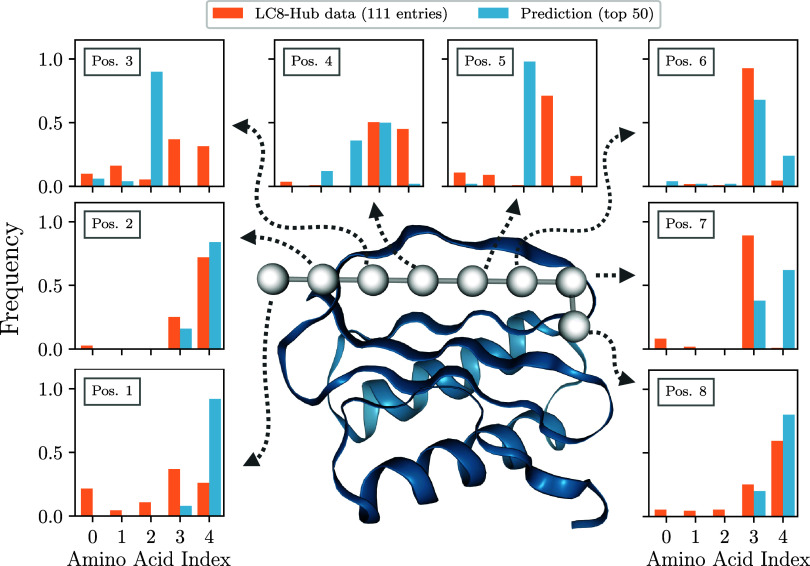
Comparison of the top 50 generated binders with 111 experimentally known binders of the LC8 hub protein. The eight histograms correspond to the eight positions in the anchor motif of LC8. For each position, the frequency of amino acid types in the generated binders (blue) and in the LC8 hub^41^ (orange) is displayed. The amino acids have been clustered into five groups, as described in “Designing a simplified coarse-grained model”.

To check that the observed correlation between the predicted and the observed sequences was not biased by the specific choice of the reduced alphabet size, in Figure S1 of the SI, we report the results of an analog analysis in which the amino acids were grouped into *D* = 10 families. As in the previous case, we observed an overall positive qualitative correlation. Similar to the results for 5 families, at positions 1, 2, 7, and 8, the amino acid family that is most frequently occurring in the experimental data set is among the two most frequently predicted.

### Classical vs Quantum Optimization

The QUBO encoding enables us to resort to a classical optimizer or quantum annealing machine to solve the optimization steps of our peptide design algorithm. A key question to address is whether, for this specific QUBO problem, existing quantum annealing machines can compete with an industry-grade optimizer on a classical computer. To address this issue, we designed 10-amino-acid-long peptides for a protein–peptide complex investigated in the previous section (PDB entry: 3BRL) using D-Wave’s hybrid classical/quantum solver (with default parameters) and Gurobi’s classical optimization algorithm.^43^ For this benchmark, we focus on sequence and structural optimization using *D* = 5 chemical species and the same parameters as in the structure-based validation. Our prescription to place the grid resulted in lattices with approximate dimensions of *L*_*x*_, *L*_*y*_ ∼ 3, and *L*_*z*_ ∼ 10. In our application to 3BRL, we used 1814 binary variables, in accordance with our estimate from eq 22.

To compare the quality and efficiency of the classical and quantum optimization algorithms, in Figure 6, we report both the lowest and average minimum-energy value (MEV) attained by classical and quantum optimization. The results of Gurobi (version 11.0.1) were obtained by performing 1000 independent runs lasting between 3 and 400 s. D-Wave’s results were obtained with 300 independent 5 s runs of the hybrid classical-quantum solver (hybrid_binary_quadratic_model_version2, with the quantum part executed on the performance-updated Advantage_system). These curves show that the two approaches yield a very similar lowest MEV and that Gurobi stops improving on the lowest MEV after running for about 10 s. However, to yield an average MEV lower than that generated by D-Wave, our Gurobi simulations need to run for approximately 200 s.

**Figure 6:**
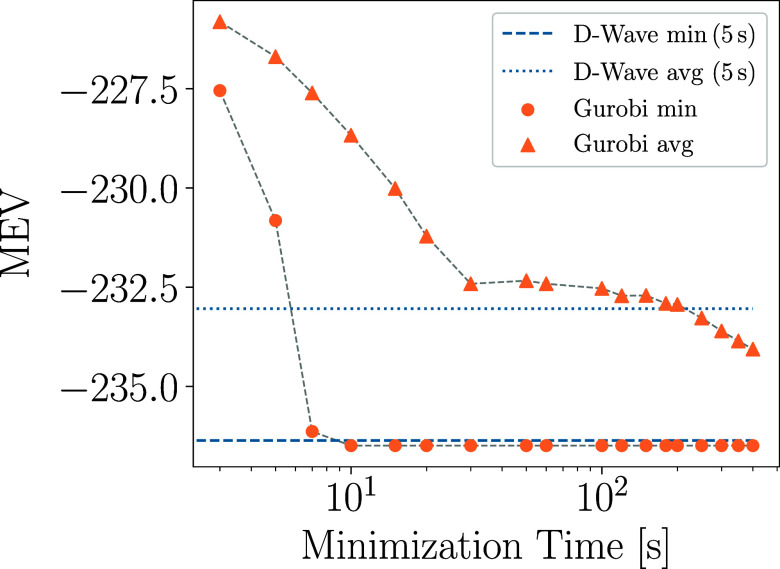
MEV obtained generating binders for the PDB entry 3BRL using classical and quantum optimization. Orange points: Lowest (circles) and average (triangles) MEV obtained in 1000 Gurobi runs as a function of the runtime of an individual simulation. Horizontal blue lines: Lowest (dashed) and average (dotted) MEV obtained in 300 D-Wave runs with 5 s of hybrid annealing time. For a fair comparison, when running classical minimization with Gurobi, we encoded the conditions imposed by *H*_anc_, *H*_occ_, and *H*_path_ as hard constraints.

Designing a chemically diverse set of hits is important for efficient drug development. In our approach, this is obtained if the optimization does not yield a single MEV but rather a distribution of diverse results peaked around a low average MEV. In the left panel of Figure 7, we compare the distributions of MEVs obtained using D-Wave and Gurobi. We note that the quantum annealer yields a continuous energy spectrum of MEVs, while the distributions generated with Gurobi are peaked in a few isolated bins. By direct inspection, we found that each solution of the annealer corresponds to a different primary sequence.

**Figure 7:**
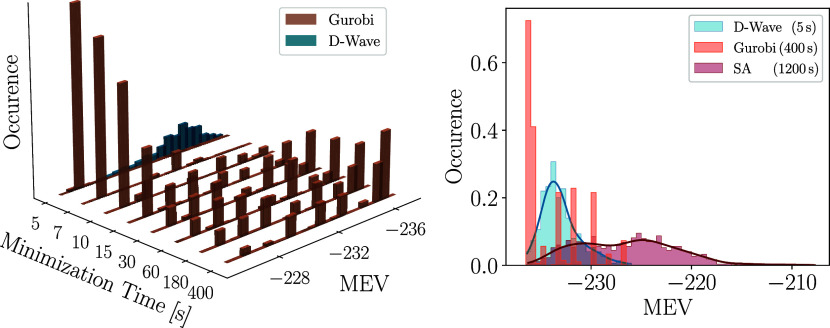
Spectrum of MEV obtained in classical and quantum optimization. Left panel: Spectra obtained in 1000 Gurobi runs with different minimization times (brown) and in 300 5-s-long runs of the D-Wave hybrid. Right panel: Spectra obtained with Gurobi run, with classical simulated annealing (SA), and with D-Wave’s hybrid solver.

Interestingly, running Gurobi longer does not lead to the discovery of more sequences. Instead, it only enhances the relative occurrence of the low-MEV sequences already discovered in shorter runs. Simulated annealing represents a conventional classical optimization scheme that may be more apt to generate a continuum spectrum of MEVs. On the right panel of Figure 7, we compare the spectra of MEVs generated by D-Wave and Gurobi with the results of 1000 independent simulated annealing runs (using the SimulatedAnnealingSampler from the Python module neal). Each consisted of 10^7^ sweeps and lasted approximately 1200 s on a desktop computer. As expected, the MEVs obtained by these relatively long simulated annealing runs are distributed according to a continuum spectrum. However, their average MEV is significantly higher than that obtained with quantum annealing runs lasting just 5 s. Collectively, this spectral analysis suggests that quantum optimization provides a promising approach to combine the request of a high binding affinity (i.e., a low MEV) and chemical diversity.

Our findings suggest that, even with the current limitations of quantum annealers, it is feasible to apply them to nontrivial peptide design tasks. The generated sequences are chemically plausible, and the optimization performance is on par with that of modern classical solvers run on standard hardware. However, it should be emphasized that a quantitative assessment of these performances is not straightforward and is far beyond the scope of the present work. Indeed, on one hand, the efficiency of the classical optimization step may be improved by resorting to more powerful computing resources. On the other hand, the quantum annealer’s performance may be improved by tuning the internal parameters of the hybrid solver, such as the annealing time and schedule.

As a final remark in this section, we stress that a potential advantage of the quantum annealing approach, relative to conventional simulated annealing schemes, is that the result does not depend on an initial guess of the solution. Indeed, at each call of the quantum optimizer, all qubits are initially prepared to be eigenstates of the σ̂_*x*_ Pauli operator, i.e., in a linear superposition of the |0⟩ and |1⟩ states. This uniform initialization avoids biasing toward specific structures and sequences. After the adiabatic switching procedure, the annealer’s wave function collapses to a single classical state that encodes a specific peptide’s sequence and structure, avoiding the resort to an initial guess.

### Improvements Required for Accurate Peptide Design

While the current version of our design algorithm can capitalize on the potential of quantum technologies in tackling the exploration problem, several improvements are needed to reach an accuracy comparable to those of state-of-the-art algorithms based on classical computing, such as BindCraft^20^ or PepInvent.^44^

First and foremost, the current algorithm ignores the conformational dynamics of the ligand and the target (Figure 8A). This shortcoming prevents, e.g., accounting for alternative target conformations, such as those generated by the induced-fit mechanism. A possible strategy to overcome this limitation consists of introducing an additional physical modeling layer in our multiscale approach (Figure 8B): In particular, the designed sequence and structure obtained after the combinatorial optimization step should be regarded as the starting point of a Metropolis Monte Carlo simulation in which the trial moves involve updating the protein and the target conformations as well as the peptide’s amino acid sequence. This procedure would in principle enable the identification of sequences that bind to non-native target conformations. A suitable physical model for this Monte Carlo calculation is the one originally developed by Kim and Hummer,^27^ which has been specifically designed to describe protein–protein interactions and has been shown to aptly account for non-native interactions in protein folding processes.

**Figure 8:**
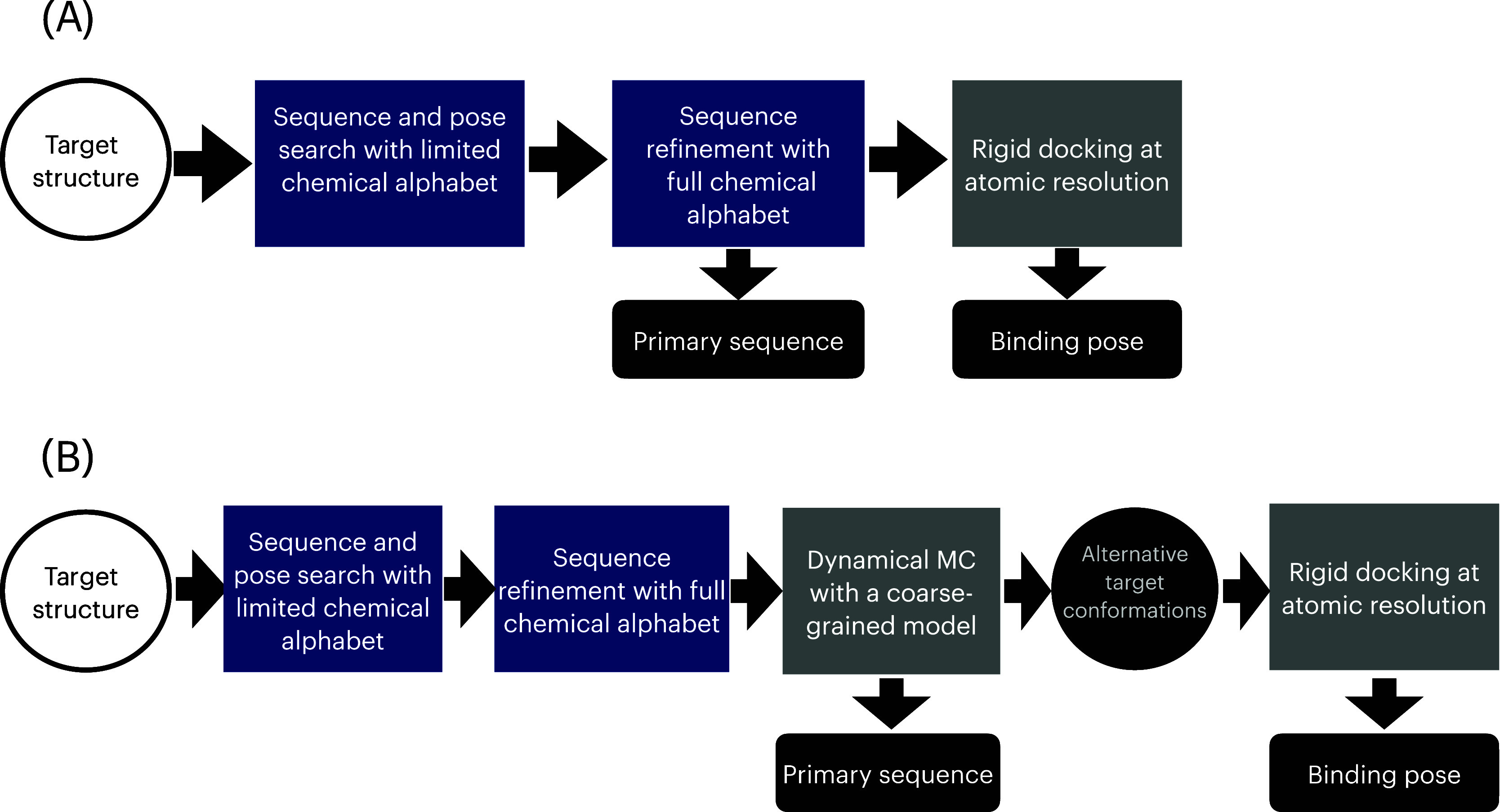
Comparison between the present version of the design algorithm (upper panel), with a possible extension that accounts for the conformational dynamics of the target and ligand (lower panel). Blue rectangles denote calculations that may be performed with a quantum optimizer. Gray rectangles are to be carried out on a classical computer. Outputs of the algorithm are highlighted in black.

One could also consider adding receptor flexibility by introducing qubits that represent the receptor and modeling the pocket using a term analogous to that in eq 18. However, this approach not only increases the qubit count due to the introduction of a “receptor grid”, but also requires preserving the identity of the receptor’s primary amino acid sequence. A naïve implementation would result in higher-order terms that scale with the number of flexible residues, thereby increasing the computational load on quantum hardware and highlighting the need for further developments in quantum encodings.

An important direction for future improvement would be to adopt more detailed representations of ligand conformation, such as encoding individual rotamers of each amino acid using dedicated qubits or introducing explicit side-chain degrees of freedom. Both approaches require a significant number of additional qubits, with the latter also requiring finer and potentially more topologically complex lattices.

## Discussion

The rapid development of quantum computing hardware raises the question of whether this emerging technology could accelerate computer-aided drug discovery. Pioneering applications of quantum algorithms to tackle drug discovery-related tasks include algorithms for molecular docking,^45–48^ solvent configuration prediction,^49^ sampling rare conformational protein transitions,^50^ protein folding,^51–55^ and protein design.^56–58^ Recently, Vakili et al. combined classical and quantum neural networks to identify small molecules that inhibit KRAS proteins.^59^

In this work, we developed a physics-based multiscale approach to *de novo* peptide design that exploits the potential of quantum hardware to enhance the simultaneous exploration of all possible peptides’ sequences and conformational states. We derived its quantum encoding starting from a rigorous statistical mechanical formulation, condition eq 2, by applying a leading-order cumulant expansion, condition eq 4, and a mean-field approximation, eq 11. Our scheme also resorts to classical computing to improve the structural resolution of the binding pose and yield atomically resolved off-lattice predictions.

We illustrated this approach with several applications, comparing our results to experimentally characterized protein–peptide complexes. As an initial illustrative toy model, we demonstrated that the algorithm generates coarse-grained peptides consistent with the expected behavior of the HP model. We then carried out two validation studies: First, we assessed the reliability of our structural predictions by comparing the binding poses of designed peptides to those obtained by redocking the peptides present in the corresponding experimentally resolved complexes. Second, we statistically compared the sequences generated by our algorithm for binding to the protein LC8 with those from a data set of experimentally verified peptide binders. Our results suggest that the algorithm successfully generates molecules with the desired structural and chemical properties. We also identify key improvements needed to enhance its reliability and bring its performance closer to that of state-of-the-art design algorithms.

In future work, it will be important to perform direct experiments aimed at assessing the binding affinity of the predicted sequences to the target protein. Other relevant improvements include increasing the algorithm’s resolution, explicitly modeling the receptor to capture mechanisms such as induced fit, generalizing the peptide binder design scheme to small molecules, and accounting for ADMET properties in the optimization process.

We compared the solutions to our design problem obtained using the D-Wave quantum annealer with those obtained using conventional simulated annealing and Gurobi, an industry-grade classical optimizer. Even using modest qubit resources, D-Wave in a few seconds generated sequences with MEVs close to those obtained with Gurobi on a desktop computer and significantly lower than those generated with much longer simulated annealing runs. We found that quantum optimization yields a continuum spectrum of MEVs, while Gurobi tends to systematically converge toward a discrete set of solutions. Direct inspection revealed that all 300 MEVs obtained with D-Wave corresponded to different peptide sequences. Therefore, the results obtained by quantum optimization combine a good predicted affinity with a good chemical diversity. Overall, our results show that even in their current early stage of development, quantum computers can already generate diverse and chemically plausible peptide binders, suggesting that they could become a valuable tool for physics-based drug design.

Our classical simulations were conducted on a desktop computer and can be sped up using more powerful computing resources. However, classical algorithms and hardware are already highly optimized, and state-of-the-art solvers like Gurobi do not profit significantly from GPU acceleration. In contrast, quantum technologies are still in their infancy. If quantum hardware continues to improve over the next several years, quantum optimization algorithms may enable tackling complex *de novo* drug design problems that remain challenging for classical machines. Furthermore, similar quantum-empowered physics-based design approaches may be developed for *de novo* design applications beyond drug discovery, such as the design of organic semiconductors or molecular nanosensors.

## Methods

### Classical and Quantum Algorithms for QUBO

The QUBO problem defined above can be solved by using classical and quantum hardware. Classical optimization schemes may combine heuristic global search algorithms (such as simulated annealing) with local refinements. Other classical optimization methods, such as the branch-and-bound algorithm used in the Gurobi optimizer,^43^ provide a more systematic exploration of the search space.

Alternatively, QUBO problems may be tackled using quantum hardware, which capitalizes on quantum superposition to enhance the exploration of the configuration space.

To solve the peptide design problem, in this study, we resorted to both classical and quantum optimizers, comparing the results obtained using Gurobi (a state-of-the-art solver widely used in academic and industrial research) and the D-Wave quantum annealer. In particular, to implement our QUBO problem on the D-Wave quantum annealer, it is convenient to recast Hamiltonian *H* in the form of a (classical) generalized Ising model. To this end, we apply the transformation σ_*l*_^*z*^ = 2*q*_*l*_ – 1, where the label *l* runs over all binary variables entering the QUBO Hamiltonian. The resulting generalized Ising Hamiltonian contains both quadratic and linear terms, i.e., it takes the form *H*_Ising_ = ∑_*l*_ *h*_*l*_σ_*l*_^*z*^ + ∑_*l*>*m*_ *J*_*lm*_σ_*l*_^*z*^ σ_*m*_^*z*^. The classical Hamiltonian is then promoted to a quantum Ising Hamiltonian by replacing each spin variable with a Pauli-*z* operator, σ_*l*_^*z*^ → σ̂_*l*_^*z*^. The eigenstates of the σ̂_*l*_^*z*^ operators are identified as the qubits of the quantum computer.

In this quantum encoding, our peptide design problem is mapped onto finding the ground state of a generalized quantum Ising Hamiltonian *Ĥ*_Ising_. This task is conveniently tackled by resorting to the so-called adiabatic switching procedure:^60^ The quantum computer’s wave function is initialized in the ground state of a Hamiltonian that is easy to solve and does not commute with σ̂^*z*^, for example, *PĤ*_in_ = −*h*∑_*l*_σ̂_*l*_^*x*^, where *h* is an arbitrary real constant. Then, the quantum annealer’s wave function is evolved for a time *t*_f_ according to the time-dependent Hamiltonian *Ĥ*(*t*) = *a*(*t*)*Ĥ*_in_ + *b*(*t*)*Ĥ*_Ising_. The so-called scheduling functions *a*(*t*) and *b*(*t*) are defined in such a way that *Ĥ*(*t*) switches from *H*_in_ to *Ĥ*_Ising_ over the time interval *t*_f_, i.e., *a*(0) ≫ *b*(0) and *a*(*t*_f_) ≪ *b*(*t*_f_). The adiabatic theorem ensures that if the sweeping process is performed sufficiently slowly compared to the minimal energy gap Δ*E* encountered (i.e., if $t_{f}\gg \frac{\hbar }{\Delta E}$ ), then the system remains in its instantaneous ground state. That is, measuring the qubits in the final quantum state yields a solution to the QUBO problem.

In our applications to realistic design problems, we resorted to the hybrid solver of the D-Wave quantum annealing machine, which combines quantum annealing with classical pre- and postprocessing steps.^61^

## Supplementary Material





## Notes

Details on the methods and the validation methods (ZIP)
The data and the corresponding scripts to generate the figures presented in this work (PDF)
