# Supplementary material for: De Novo Design of Protein-Binding Peptides by Quantum Computing
Source: J Chem Theory Comput. 2025 Sep 29;21(19):9993–10005. doi: 10.1021/acs.jctc.5c00768 (PMC12529961; doi:10.1021/acs.jctc.5c00768)
Supplement: Supplementary file 2 [file ct5c00768_si_002.pdf]

# Supplemental information for *De Novo* Design of Protein-Binding Peptides by Quantum Computing

Lars Meuser,[†,‡] Alexandros Patsilinakos,[‡] and Pietro Faccioli[*,†,¶]

†*Università di Milano-Bicocca, Dipartimento di Fisica, Piazza della Scienza 3, 20126 Milano, Italy*

‡*Sibylla Biotech S.p.A., Via Lillo del Duca 10, 20091 Bresso, Italy*

¶*INFN Sezione di Milano-Bicocca, Piazza della Scienza 3, 20126 Milano, Italy*

E-mail: pietro.faccioli@unimib.it

## Supplementary Methods

This Supplementary Information details the implementation of our design scheme, its quantum encoding, and the statistical analysis we performed to assess its reliability.

In particular, the section "Estimating of the number of contacts per amino acid" describes our method for computing the number of contacts per amino acid, a key ingredient needed in the mean-field estimate of the $\langle U_T(\Sigma)\rangle$ term of Eq. 4 of the main text. The section "Scaling of the number of qubits" reports an estimate of the number of qubits required to generate peptides using our algorithm, with respect to the grid size and the number of chemical entities. Furthermore, "Details on the structure-based validation" and "Additional results on the sequence-based validation" support the analysis reported in the main text.

## Estimating of the number of contacts per amino acid

In Eq. 11 of the main text, we estimate the average interaction of the peptide with the protein surface. In particular, for each amino acid in the sequence, we calculate the interaction it would form with any other amino acid on the protein surface according to the relative frequency it occurs on surfaces. The last ingredient needed to calculate the average interaction is the average number of contacts $\mathcal{N}_c$ formed by the sequence with the pocket.

According to the LJ potential introduced in Eq. 7 of the main text, the maximal interaction between a pair of amino acids $i$ and $j$ is $\varepsilon_{ij}$. We therefore define a partial contact as the fraction of the maximal interaction it forms, i.e., we obtain the partial contacts associated with a given pairwise interaction by dividing the interaction energy by $\varepsilon_{ij}$. Summing over all partial contacts and dividing by the number of amino acids in the ligand yields the average number of contacts $\mathcal{N}_c$.

We note that the number of contacts is a specific property of a given model for the amino acid interaction. For example, increasing the cutoff of the LJ potential leads to a larger $\mathcal{N}_c$.

In practice, we first performed a simulation with $\mathcal{N}_c = 0$. The number of contacts of the resulting binding pose was then used as the input to a new simulation. If the number of contacts was significantly different (roughly by 10 %), the procedure was repeated, initializing a new simulation with the updated value.

## Scaling of the Number of Qubits

In the following, we give an estimate on how many binary variables (or qubits) are needed to run our peptide design algorithm. Let $N_{\text{Lattice}}$ be the number of lattice points and $N_{\text{Degree}}$ be the degree of the lattice points, i.e., the number of bonds that can be assigned to each lattice point. For simplicity, we assume that the degree is the same for each grid point. As a bond connects two lattice points the total number of bonds is $N_{\text{Bonds,tot}} = N_{\text{Lattice}} N_{\text{Degree}}/2$. Furthermore let $D$ be the number of chemical building blocks per grid point. A set of $D$ qubits $\{q_i^{(k)}\}_{k\in\{1,\ldots,D\}}$ is associated to each lattice point $i$. This amounts to $D_{\text{tot}} = D N_{\text{Lattice}}$

binary variables, representing all building blocks on all grid points. Furthermore, for the implementation of nearest neighbour interactions on a quantum annealer, a set of ancillary variables $\{q_{ij}^{(k)}\}_{ij\in\{1,N_{\text{Bonds, tot}}\}}^{k\in\{1,\dots,D\}}$ is required which amounts to $N_{\text{Anc}} = DN_{\text{Bonds, tot}}$ extra variables. Note that for next-nearest neighbour interactions, no additional variables are needed. The total number of variables is therefore

$$\begin{aligned} N_{\text{tot}} &= D_{\text{tot}} + N_{\text{Bonds, tot}} + N_{\text{Anc}} \\ &= N_{\text{Lattice}} \left( D + \frac{N_{\text{Degree}} + DN_{\text{Degree}}}{2} \right) \end{aligned} \tag{S1}$$

In the case of a cubic lattice of dimensions $L_x, L_y, L_z$ the precise resource requirements amount to

$$\begin{aligned} D_{\text{tot}} &= DL_xL_yL_z \\ N_{\text{Bonds, tot}} &= (L_x - 1)L_yL_z + L_x(L_y - 1)L_z + L_xL_y(L_z - 1) \\ N_{\text{Anc}} &= N_{\text{Bonds, tot}}D \\ N_{\text{tot}} &= N_{\text{Bonds, tot}}(D + 1) + DL_xL_yL_z \end{aligned} \tag{S2}$$

In our implementation, the choice of hardware was D-Wave's quantum annealing platform. However, we could have also opted for gate-based quantum computers, optimizing the energy using, e.g., the quantum approximate optimization algorithm[1] or variational quantum eigensolvers.[2] Although currently available quantum annealers have more qubits than gate-based quantum computers, implementing the algorithm on a gate-based quantum computer requires fewer qubits. In particular, the ancillary qubits implementing the nearest neighbour interactions are not required on a gate-based quantum computer. We could further reduce the number of lattice qubits using a logarithmic encoding from $N_{\text{Lattice}}$ to $\log(N_{\text{Lattice}} + 1)$.

We would therefore require

$$\begin{aligned} N_{\text{BB, tot}} &= L_x L_y L_z \log_2(D+1) \\ N_{\text{Bonds, tot}} &= (L_x - 1)L_y L_z + L_x(L_y - 1)L_z + L_x L_y (L_z - 1) \\ N_{\text{tot}} &= N_{\text{Bonds, tot}} + \log_2(D+1) L_x L_y L_z \end{aligned} \tag{S3}$$

qubits on a gate-based quantum computer. However, the number of qubits is not the only relevant measure in terms of resource requirements. For example, a logarithmic encoding introduces higher-order interaction terms, making the implementation of the Hamiltonian more involved.

## Details on the structure-based validation

In the main text, we described a structural validation of design algorithm. Note that this analysis was performed by resorting to local docking. This might explain the high scores associated with some of the randomly generated peptides. However, in the protein-peptide complexes we considered, global docking did not necessarily yield good results, even for the redocking experiments, so it could not be used to validate our algorithm.

Using the recommended simulation parameters (3 million steps per amino acid and 200 independent runs) each local redocking calculation took roughly 3 hours on a desktop computer.

## Additional results on the sequence-based validation

In the main text, we generated ligands for the LC8 hub protein and compared their sequences to sequences of known binders. We clustered the amino acids into five families of similar types before comparing the relative occurrences at each position of the LC8 binding motif, as shown in Fig. 5 of the main text.

In Fig. S1, we present similar results obtained by clustering the amino acids into 10

families. Even at this finer resolution, we still find that in four out of eight positions, namely, in positions 1, 2, 7 and 8, the most frequently experimentally occurring amino acid type is among the two most frequently predicted ones. In position 4, the most frequently predicted amino acid type is the second most occurring one.

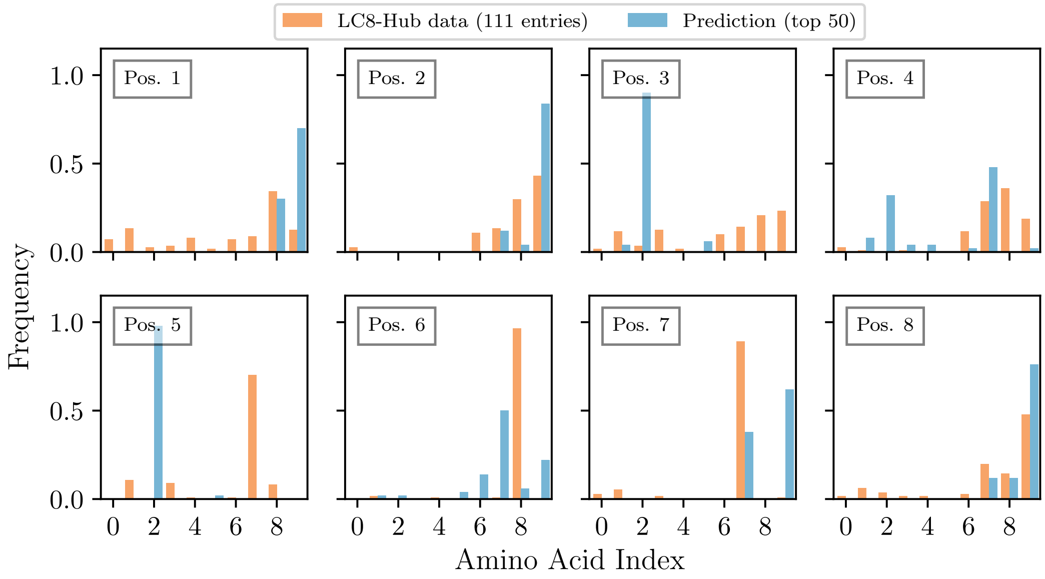


Figure S1: Comparison of proposed generated binders and known binders of the LC8 hub protein as in Fig. 5. The amino acids have been clustered into 10 amino acid types. In positions 1, 2, 4, 7, and 8, our algorithm proposes amino acids of similar type to those observed in the experimental dataset.

## References


(1) Farhi, E.; Goldstone, J.; Gutmann, S. A Quantum Approximate Optimization Algorithm. 2014; `http://arxiv.org/abs/1411.4028`.

(2) Peruzzo, A.; McClean, J.; Shadbolt, P.; Yung, M.-H.; Zhou, X.-Q.; Love, P. J.; Aspuru-Guzik, A.; O'Brien, J. L. A variational eigenvalue solver on a photonic quantum processor. *Nature Communications* **2014**, *5*, 4213.
